# A Brief Report on a Reduced Preference for Passive-Avoidant Leadership After a Restless Night

**DOI:** 10.3389/fpsyg.2018.01888

**Published:** 2018-10-02

**Authors:** Morten Nordmo, Olav Kjellevold Olsen, Ragna Rosseland, Tone Fidje Blågestad, Ståle Pallesen

**Affiliations:** ^1^Department of Psychosocial Science, University of Bergen, Bergen, Norway; ^2^Department of Clinical Psychology, University of Bergen, Bergen, Norway

**Keywords:** sleep, leadership, followership, sleep fragmentation, passive avoidant leadership

## Abstract

The purpose of this study is to investigate the effect of fragmented sleep on followers’ leadership preferences. In a counterbalanced experimental study involving 39 followers, changes in leadership preferences were recorded after one night of fragmented sleep (awakened every 80 min during the night), compared to a rested condition with the conditions separated by seven nights. The results showed that the participants rated passive-avoidant leadership less ideal, after one night of fragmented sleep. No differences regarding preference for transactional or transformational leadership occurred. Thus, negative perceptions of leaders may partly stem from reduced sleep patterns. However, further studies are needed to confirm this finding.

## Introduction

Recently, there has been increasing interest in the effect of reduced sleep on leadership behavior and effectiveness. Two experimental studies suggest that leadership is impaired by sleep loss (Barnes et al., 2015; Olsen et al., 2016). Olsen et al. (2016) found that long-term partial sleep deprivation resulted in a decrease in transactional and transformational leadership, and a large increase in passive-avoidant leadership. In a similar vein, Barnes et al. (2015) found that reduced sleep quality was associated with an increase in abusive supervision. These findings would arguably better inform the leadership process if we also knew how reduced sleep affects followers. No experimental study has investigated the effect of sleep loss on followers’ leadership preferences. The lack of understanding of the effects of reduced sleep in followers is noteworthy given the likelihood of both leaders and followers being sleep deprived. Presumably, there are many situations where both are sleep deprived. In this scenario, there may be a significant negative interaction between the heightened needs of followers and the reduced leadership capabilities, and changing leadership styles (Olsen et al., 2016). The full range of leadership model (FRLM) (Bass and Riggio, 2006) as well as other leadership models suggests that effective leadership varies across different circumstances where leaders ideally should able to adapt their behavior to the specific context (House, 1996). Leadership adaptation may also be appropriate in contexts where teams of worker experience sleep restrictions. In this brief research report, we present evidence that suggests reduced sleep may affect leadership preferences. The main finding, a reduced preference for passive-avoidant leadership after poor sleep, may shed light on the negative consequences of reduced sleep in the leader-follower dynamic.

## Materials and Methods

### Sample

The data from this study stems from an experimental investigation of the relationships between sleep, emotions, and pain. Participants were recruited via email and at lectures at the University of Bergen. The sample is comprised of students. However, psychology students were prohibited to participate in the study. The sample originally consisted of 40 participants, but was reduced to 39 (*N* = 39) as one of the participants did not show up for testing on the first day and was excluded from the study. The final sample consisted of 18 men and 21 women, ranging from 19 to 26 years old (*Mean* = 22.10, *SD* = 1.76). All participants signed informed consent forms and were screened for self-reported health problems. The participants received 500 nok. (approximately 63 USD) at the end of the study. This project was approved by a regional ethics committee.

### Procedure

We instructed the participants to sleep as usual 5 days before being tested in the two experimental conditions. We used sleep diaries, kept by the participants, to verify their sleep in this 5-day period. On both experimental and control nights the participants were instructed to go to bed at 22:30 and to spend a total of 8 h in the bed. The control condition involved no disturbances. In the experimental condition, we woke the participants with a telephone call, six times (every 80 min). At each wake-up call, the participants were instructed to complete a series of online questionnaires that approximately took about 10 min to complete at each awakening. The timing and duration of the awakenings were verified by the collected data. In order to be included in the present study, the participants needed to respond accordingly to at least five of the six wake-up calls. After the night of normal sleep and of fragmented sleep, we asked the participants to describe an ideal leader, using the multifactor leadership questionnaire (MLQ), for a challenging and difficult task (**Appendix 1**). The hypothetical task involved a high-risk rescue operation in difficult conditions. Thus, the participants completed the description of an ideal leader two times, one in a rested state and one in a fragmented sleep state. The two conditions were separated by 7 days of normal sleep.

### Measures

The MLQ 5X (Bass and Avolio, 1995) was used to define and measure the participants’ description of an ideal leader and compromises the dependent variable. The questionnaire contains 36 items describing behavior and attributes, each rated on a five-point scale ranging from zero to four (0 = rarely; 4 = to a large extent). In the present study, three indexes were extracted in both conditions: Transformational leadership when sleep fragmented (TL: α = 0.875), and rested (TL: α = 0.842); Transactional leadership when sleep fragmented (TR: α = 0.706), and rested (TR: α = 0.640); Passive-avoidant leadership when sleep fragmented (PA: α = 0.555) and rested (PA: α = 0.525).

Karolinska Sleepiness Scale (KSS) was used to assess the effect of fragmented sleep in terms of sleepiness and functions as a manipulation check (Åkerstedt and Gillberg, 1990). The KKS is a state measure of subjective sleepiness. The response alternatives range from 1 to 9, where 1 represents “*Very well rested*” and 9 represents “*Very sleepy, can’t stay awake.*”

### Statistics

The three MLQ indexes and the KSS were tested for normality and homogeneity of variance. Shapiro–Wilks test revealed that transformational leadership and the KSS violated the assumption of normality. Because of this, we conducted four bootstrapped paired sample *t*-tests, and generated confidence intervals (95%), with 1000 samples, to determine if one night of fragmented sleep significantly changed the rating of an ideal leader. All test significance tests were two-tailed, and we set the significance level to 0.05. We determined effect sizes with Cohen’s d. As a benchmark, Cohen (1988) determines effects sizes (Cohen’s d) in the behavioral sciences in the following way: A value of 0.2 represents a small effect size, 0.5 a medium effect and 0.8 a large effect.

## Results

### Sleepiness

The participants were significantly less sleepy after one night of uninterrupted sleep (*M* = 3.9, *SD* = 1.83), compared one night of fragmented sleep (*M* = 5.41, *SD* = 1.87), as measured by the KKS. The mean difference (1.513, *SE* = 0.395) was significant when using a bootstrapped *t*-test (*CI* = 0.744, 2.333, *P* = 0.001), with a large effect size (*d* = 0.898). This manipulation check confirms that the experimental manipulation of sleep fragmentation had an effect on the participants’ sleepiness.

### MLQ

**Table 1** shows descriptive statistics for the main and sub-indexes in the MLQ and KSS. We found that one night of fragmented sleep did not significantly change participants’ description of an ideal leader for a transformational or transactional leadership style. However, one night of fragmented sleep did affect followers’ description of an ideal leaders’ level of passive-avoidant leadership style.

**Table 1:** Descriptive statistics for MLQ and KSS.

		Range	*M*	*SD*
TL	Fragmented	2.10	3.15	0.44
	Rested	2.15	3.09	0.44
II(A)	Fragmented	1.25	3.51	0.35
	Rested	2.25	3.41	0.55
II(B)	Fragmented	1.75	3.01	0.49
	Rested	3.00	2.85	0.60
IM	Fragmented	2.00	3.32	0.51
	Rested	2.25	3.24	0.41
IS	Fragmented	3.50	2.91	0.66
	Rested	2.75	2.93	0.59
IC	Fragmented	4.00	3.01	0.77
	Rested	3.75	2.97	0.84
TR	Fragmented	2.50	2.76	0.58
	Rested	2.13	2.68	0.55
CR	Fragmented	2.25	2.99	0.60
	Rested	2.25	2.88	0.51
MBE-a	Fragmented	3.00	2.52	0.72
	Rested	3.25	2.48	0.79
PA	Fragmented	1.38	0.58	0.37
	Rested	1.75	0.69	0.41
LzF	Fragmented	1.00	0.21	0.28
	Rested	1.50	0.24	0.36
MBE-P	Fragmented	2.00	0.94	0.58
	Rested	2.75	1.13	0.63
KSS	Fragmented	6.00	5.41	1.87
	Rested	7.00	3.90	1.83


TL, transformational leadership; TR, transactional leadership; PA, passive avoidant leadership; LzF, laizzes faire leadership; MBE-p, management by exemption passive; II(A), idealized influence attitudes; II(B), idealized influence behavior; IM, inspirational motivation; IS, intellectual stimulation; IC, individualized consideration; CR, contingent reward; MBE-p, management by exemption passive, *n* = 39.

There were significantly higher scores for passive-avoidant leadership in the rested state (*M* = 0.685, *SD* = 0.413) compared to the fragmented sleep state (*M* = 0.576, *SD* = 0.373). The mean difference (*-*0.108, *SE* = 0.049) was significant when using bootstrapped paired *t*-test (*CI* = -0.208, -0.006, *P* = 0.047). Effect size *d* = 0.34. In the rested state, the participants described the ideal leader as more passive-avoidant, compared to a fragmented sleep state.

## Discussion

The goal of this study was to investigate the effect of fragmented sleep on followers’ description of an ideal leader in an operative situation. We observed no changes in ratings for transformational or transactional leadership, but participants rated an ideal leader as less passive-avoidant when sleep fragmented. The results show that this difference is largely due to changes in preference for management-by-exception-passive. This change in preference for this leadership trait entails that sleep fragmented followers are less likely to accept a leader who only intervenes after errors or breaches of standards. This finding has implications for the effectiveness of leadership when followers are sleep deprived. It suggests that sleep fragmented followers are more likely to prefer and perform better when a leader adjusts to the situation by increasing the level of active leadership. This result mirrors how sleep deprived leaders show a significant increase in passive-avoidant leadership (Olsen et al., 2016). This interaction may be partly responsible for the relationship between sleep and dysfunctional groups (Barnes et al., 2011). This study, while useful as an early inquiry into sleep and followership should, however, be viewed with caution based on the low-reliability measure of PA, and the near zero mean scores. The low interrater reliability of passive-avoidant leadership indicates that our participants did not agree on the ideal leaders’ levels of this leadership trait. This may be due to some of the participants emphasizing the demanding leadership situation described, while others may not. The passive-avoidant leadership trait may be more ambiguous in a prototypical ideal leader, compared to when rating the effectiveness of a specific leader. Ratings of leaders’ passive-avoidant leadership behavior are unlikely to be this low. Rating of ideal leadership, without external leadership stimuli, may also be more ambiguous, compared to ratings of actual leaders. This may have contributed to lower reliability estimates. Furthermore, knowledge of being sleep fragmented may have influenced results through demand characteristics (Orne, 1962).

Some assets of the present study also deserve mention. Assessing leadership preferences was performed at the same time in both conditions, limiting differential influences from circadian factors. In addition, the present study was based on an experimental counterbalanced crossover design, ensuring high internal validity. Other strengths of the present study entail the use of objective verifications of wake during the designated wake-up calls and the use of manipulation checks. It should also be noted that the level of sleep fragmentation was rather moderate in the present study (just one night) which arguably strengthens the ecological validity of the present study.

### Further Studies

Future studies may have followers rate different sets of leaders in both sleep fragmented and rested states. The leaders would be varying in transformational, transactional and passive-avoidant behavior. The next step will be to investigate if leadership preferences translate into better performance outcomes. This could involve using confederate leaders with predetermined leadership styles and either sleepy or rested followers assessing performance, in-group dynamic and group cohesiveness. This followership centric research may yield a better understanding of leadership processes (Crossman and Crossman, 2011) in different sleep conditions.

The results of this study are confined to the conceptualized definitions of leadership within the FRLM. A broader understanding of how lack of sleep affects leadership preference should include other representations of leadership that differ from the FRLM and the MLQ. The MLQ confounds the active and positive, as well as the negative and passive elements of leadership. Measuring ideal leadership traits that are active and negative, as well as positive and passive would potentially elucidate our findings. Lack of sleep and leadership preference may interact differently when measuring other models ofleadership, such as exploitative (Schilling, 2009), autocratic (Van Vugt et al., 2004) or abusive supervision (Barnes et al., 2015).

## Ethics Statement

This study was carried out in accordance with the recommendations of Regional committees for medical and health research ethics, REC West, with written informed consent from all subjects. All subjects gave written informed consent in accordance with the Declaration of Helsinki. The protocol was approved by the REC West.

## Author Contributions

MN contributed to theory development, discussion, and statistics. OO contributed to design, discussion, and analysis. RR and TB contributed to data collection and discussion. SP contributed to data collection, design, and discussion.

## Conflict of Interest Statement

The authors declare that the research was conducted in the absence of any commercial or financial relationships that could be construed as a potential conflict of interest.

## Appendix 1 Situational description

Imagine that you and 20 of your fellow students are chosen to participate in a search and rescue operation in the mountains, north of Bergen. The weather is harsh, with high winds and temperatures below 0 degrees Celsius. Your mission is to rescue two patients who have escaped from Sandviken psychiatric hospital. When found, the patients have to be transported down from the mountain. There is a high likelihood of the patients freezing to death during the night, if not found. Therefore, time is of importance. Please also note that the patients may be paranoid and aggressive.

You will now describe what type of leader you would ideally have in this situations, given that the mission starts now. Judge how each of the statements below fits the description of a leader you want for this mission and cross in the box where you find most desirable leadership behavior for you and your group in your situation.
